# Strigolactones positively regulate defense against root-knot nematodes in tomato

**DOI:** 10.1093/jxb/ery439

**Published:** 2018-12-21

**Authors:** Xuechen Xu, Pingping Fang, Hui Zhang, Cheng Chi, Liuxia Song, Xiaojian Xia, Kai Shi, Yanhong Zhou, Jie Zhou, Jingquan Yu

**Affiliations:** 1 Department of Horticulture, Zijingang Campus, Zhejiang University, Hangzhou, P.R. China; 2 Zhejiang Provincial Key Laboratory of Horticultural Plant Integrative Biology, Hangzhou, P.R. China

**Keywords:** Abscisic acid, basal resistance, *MYC2*, root-knot nematode, *Solanum lycopersicum*, strigolactones

## Abstract

Strigolactones (SLs) are carotenoid-derived phytohormones that are known to influence various aspects of plant growth and development. As root-derived signals, SLs can enhance symbiosis between plants and arbuscular mycorrhizal fungi (AMF). However, little is known about the roles of SLs in plant defense against soil-borne pathogens. Here, we determined that infection with root-knot nematodes (RKNs; *Meloidogyne incognita*) induced SL biosynthesis in roots of tomato (*Solanum lycopersicum*). Silencing of SL biosynthesis genes compromised plant defense against RKNs, whilst application of the SL analog *rac*GR24 enhanced it. Accumulation of endogenous jasmonic acid (JA) and abscisic acid (ABA) in the roots in response to RKN infection was enhanced by silencing of SL biosynthetic genes and was suppressed by application of *rac*GR24. Genetic evidence showed that JA was a positive regulator of defense against RKNs while ABA was a negative regulator. In addition, *rac*GR24 enhanced the defense against nematode in a JA-deficient mutant but not in an ABA-deficient mutant. Silencing of SL biosynthetic genes resulted in up-regulation of *MYC2*, which negatively regulated defense against RKNs. Our results demonstrate that SLs play a positive role in nematode defense in tomato and that MYC2 negatively regulates this defense, potentially by mediating hormone crosstalk among SLs, ABA and JA.

## Teaser

Strigolactones positively regulate defense against infection by root-knot nematode in tomato through a process that is dependent on MYC2 integrating SL, ABA, and JA signaling

## Introduction

Strigolactones (SLs) are plant hormones that were first identified in 1966 as a germination stimulant in the parasitic weed genus *Striga* (Cook *et al.*, 1966). SLs are derived from carotenoids via sequential oxidative cleavage by carotenoid-cleavage dioxygenases, and thus belong to the apocarotenoid class of phytohormones, which includes abscisic acid (ABA; Matusova *et al.*, 2005; López-Ráez *et al.*, 2008). Several studies have demonstrated that *CAROTENOID CLEAVAGE DIOXYGENASE7* (*CCD7*), *CCD8*, and *MORE AXILLARY GROWTH1* (*MAX1*) are involved in the biosynthesis of SLs, whilst *MAX2* plays a role in SL perception/signaling (Gomez-Roldan *et al.*, 2008; Umehara *et al.*, 2008; Waters *et al.*, 2017). MAX2 has been shown to participate in a SKP1-CUL1-F-box-protein (SCF)-type ubiquitin ligase complex, and to catalyse the ubiquitination of specific proteins destined for proteasomal degradation (Ruyter-Spira *et al.*, 2013). SLs play pivotal roles in modulating the coordinated development of roots and shoots, in plant–microbe symbiosis, and in stress responses (Akiyama *et al.*, 2005; Gomez-Roldan *et al.*, 2008; Kapulnik *et al.*, 2011*a*; Ha *et al.*, 2014). SLs suppress lateral root primordial development and lateral root-forming potential under phosphate-sufficient conditions, whilst positively regulating the number and outgrowth of lateral roots under phosphate-limiting conditions (Ruyter-Spira *et al.*, 2011). In addition, SLs have been suggested to have a positive effect on root-hair elongation (Kapulnik *et al.*, 2011*b*). With regards to shoot architecture, a lack of SL biosynthesis or signaling components results in increased numbers of lateral shoot branches (Gomez-Roldan *et al.*, 2008; Umehara *et al.*, 2008). In addition, SLs can enhance symbiosis between plants and arbuscular mycorrhizal fungi (AMF) by inducing hyphal branching (Akiyama *et al.*, 2005). Likewise, SLs can promote rhizobium–legume symbiosis, possibly through a stimulatory effect on bacterial surface motility that thus facilitates the establishment of the symbiosis (Foo and Davies, 2011; Peláez-Vico *et al.*, 2016; McAdam *et al.*, 2017). Plants impaired in SL biosynthesis or signaling exhibit increased sensitivity to drought and salt stress, suggesting that SLs positively regulate plant responses to abiotic stress (Bu *et al.*, 2014; Ha *et al.*, 2014). SLs have also been shown to be involved in responses to biotic stress. For example, Arabidopsis SL biosynthesis and signaling mutants display enhanced symptoms when infected with the biotrophic actinomycete *Rhodococcus fascians* (Stes *et al.*, 2015). Similarly, RNAi silencing of tomato *CCD8* has been shown to result in increased susceptibility to the pathogens *Botrytis cinerea* and *Alternaria alternata*, and this is associated with decreased levels of the defense-related hormones abscisic acid (ABA), jasmonic acid (JA), and salicylic acid (SA) in leaves (Torres-Vera *et al.*, 2014).

Plant parasitic nematodes are mostly soil-dwelling microscopic worms that attack a number of important crops and are estimated to be responsible for more than $157 billion of global economic losses every year (Abad *et al.*, 2008; Holbein *et al.*, 2016). Among these nematodes, the most economically important groups are the sedentary endoparasites, which include root-knot nematodes (RKNs, *Meloidogyne* spp.). RKNs have a wide range of plant hosts and can establish feeding sites near the plant vasculature (Jones *et al.*, 2013). Several phytohormones are known to be involved in the defense against nematodes. Among them, JA and ethylene (ET) function as positive regulators, whereas ABA acts as a negative regulator (Nahar *et al.*, 2011, 2012; Kammerhofer *et al.*, 2015; Song *et al.*, 2018). The effect of brassinosteroids (BRs) on nematode infection is dependent on concentration and species (Nahar *et al.*, 2011; Song *et al.*, 2018). JA or ET application onto the shoots of rice induces a systemic defense in the roots against infection by nematodes, with the ET-induced defense involving strong activation of JA biosynthesis and signaling genes (Nahar *et al.*, 2011). By contrast, exogenous ABA treatment drastically compromises the defense of rice against nematodes (Nahar *et al.*, 2012). MYC2, a basic helix-loop-helix (bHLH) transcription factor, functions as a positive regulator of JA-biosynthesis and of JA-responsive genes such as *LIPOXYGENASE3* (*LOX3*) and *VEGETATIVE STORAGE PROTEIN2* (*VSP2*) in wound responses, and also acts as a negative regulator of JA-responsive genes such as *PLANT DEFENSE FACTOR1.2* (*PDF1.2*) and *HEVEIN-LIKE PROTEIN* (*HEL*) in defense responses (Lorenzo *et al.*, 2004). Thus, the outcome of defense against pathogens is determined by complex crosstalk among plant hormones (De Vleesschauwer *et al.*, 2014).

Although SLs are critical regulators of plant–microbe interactions in the rhizosphere, their role in plant defense against soil-borne pathogens has received little attention. The root-knot nematode *Meloidogyne incognita* is a pathogen of many economically important crops and so we used a tomato–*M. incognita* system as a model in this study. Our results demonstrate that SLs are actively involved in the regulation of plant defense against nematodes by altering the accumulation of ABA and the transcription of *MYC2*.

## Materials and methods

### Plant material and growth conditions

Seeds of the tomato (*Solanum lycopersicum*) cultivars Ailsa Craig and Castlemart, and the ABA-deficient mutant *notabilis* (*not*; Ailsa Craig background) were obtained from the Tomato Genetics Resource Center (http://tgrc.ucdavis.edu). The JA-deficient mutant *suppressor of prosystemin-mediated responses2* (*spr2*; Castlemart background) was obtained from Dr Chuanyou Li (Chinese Academy of Sciences, Beijing, China). The *spr2* mutation abolishes the function of a tomato fatty acid desaturase (now designated SlFAD7), thus resulting in defects in the octadecanoid pathway for JA biosynthesis (Li *et al.*, 2003). The *not* mutant has a null-mutation in the gene *SlNCED1*, encoding a 9-*cis*-epoxycarotenoid dioxygenase involved in ABA biosynthesis (Burbidge *et al.*, 1999). Seeds were surface-sterilized with 4% sodium hypochlorite containing 0.02% (v/v) Tween-20, rinsed thoroughly with sterile water, and then put on moistened filter paper at 28 °C in darkness for 48 h. The germinated seeds were subsequently sowed in pots (diameter 6 cm, depth 8 cm) filled with steam-sterilized sand and vermiculite (1:1, v/v). The plants were cultivated in a growth chamber with 14/10 h light/dark cycle at 25/20 °C, and 600 μmol m^−2^ s^−1^ photosynthetic photon flux density (PPFD), and fertilized with Hoagland’s nutrient solution.

Tobacco rattle virus (TRV)-based virus-induced gene-silencing (VIGS) was used to repress the target genes. Tomato seedlings at 2 weeks old that had a pair of newly emerged true leaves were infiltrated with TRV constructs and maintained in the growth chamber at 21/19 °C until nematode inoculation (Liu *et al.*, 2002). The cDNA fragments of *CCD7*, *CCD8*, *MAX1*, *PI-1*, *PI-2*, and *MYC2* were PCR-amplified using gene-specific primers (Supplementary Table S1 at *JXB* online). qRT-PCR was performed to determine the gene-silencing efficiency. As a positive control the tomato phytoene desaturase (*PDS*) gene was silenced using a previously described pTRV-*PDS* construct (Liu *et al.*, 2002). After 2–3 weeks, when the pTRV-*PDS* plants showed leaf photobleaching symptoms, the plants were inoculated with nematodes and maintained at 25/22 °C in a growth chamber until sampling.

For the *rac*GR24 soil-drenching treatment, 24 h before nematode inoculation or sample collection, the roots of tomato plants at the four-leaf stage were drenched with different treatment solutions. A stock solution of the synthetic analog *rac*GR24 (Chiralix, Nijmegen, the Netherlands) at 25 mM was prepared by dissolving in acetone. *rac*GR24 was then diluted with distilled water to 1, 3, and 9 µM solutions. Distilled water with the same amount of acetone was used as the control. Each plant was drenched with 5 ml of solution 24 h before RKN infection. After nematode infection, each plant was drenched with a *rac*GR24 solution twice per week until sampling.

### RKN infection and susceptibility assays

RKNs (*Meloidogyne incognita*, rac1, provided by Dr Deliang Peng from the Chinese Academy of Agricultural Sciences, Beijing, China) were cultured on tomato plants (*S. lycopersicum* cv. Moneymaker) grown on sand and vermiculite (1:1, v/v) at 22–26 °C in a greenhouse. Nematodes were extracted from 3-month-old infected plants according to the method described by de Ilarduya *et al.* (2001) with minor modifications. Briefly, eggs were extracted from infected roots by processing in 0.52% sodium hypochlorite in a blender for 2 min at high speed (Hussey and Barker, 1973). Eggs and root debris were collected using a 500-mesh sieve. Second-stage juveniles (J2s) were obtained by hatching the eggs in a modified Bearmann funnel, in which wire-mesh baskets were lined with two layers of paper towels, set in a glass Petri dish; the funnel was filled with the egg mixture and then incubated at 25 °C. J2s were collected after 4 d and used immediately. The content of J2s in the solution was determined using a microscope (DM4000B; Leica).

Tomato plants at the four-leaf stage were inoculated with 1000 *M. incognita* J2s per plant in 5 ml of water, applied with a pipette over the surface of the soil around the primary roots. The plants were then maintained in a growth chamber for 4 weeks. All sand/vermiculite particles were then washed from the roots, after which the fresh root weight of the plants was measured. To visualize the galls, roots were boiled for 3 min in 0.8% acetic acid and 0.013% acid fuchsin. After washing with running tap water, roots were destained in acid glycerol. Nematode susceptibility was evaluated by counting the number of galls per plant and calculating the number of galls per unit weight of fresh roots (Nahar *et al.*, 2011).

### Purification of root extracts and germination bioassays

Root extracts were purified according to the method described by López-Ráez *et al.* (2008) with minor modifications. Briefly, frozen lateral roots (0.5 g) from 3–4 plants were ground in a mortar filled with liquid nitrogen and then extracted with 2 ml of ethyl acetate in a 10-ml disposable tube. After being vortexed, the homogenate was shaken at 4 °C overnight. Samples were then centrifuged at 4000 *g* for 10 min at 4 °C using a Centrifuge 5810R (Eppendorf). The organic phase was collected, and the remaining pellets were re-extracted with another 2 ml of ethyl acetate for 1 h, after which they were centrifuged. The combined ethyl acetate fractions were dried under a flow of N_2_ gas. The residue was dissolved in 1 ml of 60% acetone/water (v/v) and stored at –20 °C until use in the germination bioassays: the ethyl acetate was removed from the samples under a flow of N_2_ gas before the bioassays. In each experiment, the extracts were diluted to the same ratio of root fresh weight per milliliter of root extract before analysis.

Germination bioassays were conducted as described by Matusova *et al.* (2005) with slight modifications. *Phelipanche aegyptiaca* seeds, which were collected in Xinjiang, China in 2015, were kindly provided by Dr Jinxia Cui (Shihezi University, Xinjiang, China). Preconditioning and germination assays were performed under sterile conditions. The seeds were surface-sterilized in 2% sodium hypochlorite containing 0.02% (v/v) Tween-20 for 5 min, after which they were rinsed thoroughly with sterile distilled water. Approximately 300–400 seeds were spread on a glass-fiber filter paper disc (diameter 2 cm) and placed into sterile Petri dishes (diameter 3 cm) lined with two layers of Whatman filter paper wetted with 0.8 ml of sterile distilled water. The Petri dishes were sealed with medical air-permeable adhesive tape and incubated for preconditioning. The *P. aegyptiaca* seeds were preconditioned at 21 °C in darkness for 1 week. The dishes were checked regularly, and water was added as required. Aliquots (300 µl) of root extract were added to three Petri dishes containing preconditioned seeds. The synthetic germination stimulant *rac*GR24 (10^–9^ M) and distilled water were included in each bioassay as positive and negative controls, respectively. After 7 d, the number of germinated seeds was counted with the aid of a microscope (DM4000B; Leica). Seeds with a protruded radicle were considered as germinated.

### Measurement of phytohormones

For measurement of SL, root extracts were purified and measured according to the method of Ruiz-Lozano *et al.* (2016) with modifications. Frozen roots (0.5 g) were ground in a mortar filled with liquid nitrogen and then extracted with 2 ml of 40% acetone/water in a 10-ml disposable tube. After being vortexed, the homogenate was centrifuged at 12 000 *g* for 5 min at 4 °C using a Centrifuge 5810R (Eppendorf). The liquid was discarded, after which the remaining solids were eluted with 2 ml of 50% acetone/water and centrifuged at 12 000 *g* for 5 min at 4 °C. The supernatant was stored at –20 °C until use. The quantification of SLs was performed using ultra performance liquid chromatography coupled to tandem mass spectrometry (UPLC-MS/MS; Varian 320-MS LC/MS, Agilent Technologies) as described previously (Koltai *et al.*, 2011). The peak areas represented the SLs levels.

For measurements of JA and ABA, lateral roots were sampled 1 d after infection with *M. incognita* according to the method of Wu *et al.* (2007). Phytohormone extraction and analysis were performed as previously described (Wang *et al.*, 2016). Briefly, 100 mg of frozen root material was homogenized in 1 ml of ethyl acetate that had been supplemented with D_5_-JA and D_6_-ABA (C/D/N Isotopes Inc, Canada) as internal standards at a final concentration of 100 ng ml^−1^. The homogenate was shaken in darkness at 4 °C overnight. After being centrifuged at 18 000 *g* for 10 min at 4 °C, the supernatant (ethyl acetate phase) was collected, and the pellet was re-extracted with another 1 ml of ethyl acetate and centrifuged. The combined supernatants were dried under a flow of N_2_ gas. The residue was then resuspended in 0.5 ml of 70% (v/v) methanol and centrifuged at 18 000 *g* for 2 min at 4 °C, and the supernatants were analysed by using UPLC-MS/MS on an Agilent 1290 infinity HPLC system (including a vacuum degasser, a binary pump, a column oven, and an autosampler) coupled to an Agilent 6460 Triple Quad LC-MS device. The parent ions, daughter ions, and collision energies used for these analyses are listed in Supplementary Table S2.

### Total RNA extraction and gene expression analyses

Total RNA was extracted from 100 mg of tomato root or leaf tissue using a total RNA kit (Omega Bio-tek, Inc., Georgia, USA) in accordance with the manufacturer’s instructions (genomic DNA was removed). A sample of 1 μg total RNA was reverse-transcribed to synthesize cDNA using a HiScript QRT SuperMix Kit (Vazyme Co., Nanjing, China). qRT-PCR was performed using SYBR Green PCR Master Mix (Vazyme Co.) on a StepOnePlus Real-time PCR Detection System (Applied Biosystems). The specific primers used for qRT-PCR are listed in Supplementary Table S3. The PCR protocol was as follows: denaturation at 95 °C for 5 min followed by 40 cycles of denaturation at 95 °C for 10 s, annealing at 58 °C for 30 s, and extension at 72 °C for 10 s. At the end of each PCR cycle, a dissociation curve was generated using software provided with the StepOnePlus Real-time PCR Detection System to verify that a single product was amplified. Three biological and three technical replicates were used to determine the mRNA expression level of the target gene, and the generated threshold cycle (*C*_T_) was used to calculate transcript abundance relative to that of the housekeeping gene *Actin* (Mascia *et al.*, 2010). The mRNA quantification procedure was based on the method of Livak and Schmittgen (2001).

### Statistical analyses

Statistical analysis was performed using ANOVA followed by Duncan’s multiple range test (*P*<0.05). For the determination of nematode susceptibility, 10 plants constituted one replicate per treatment. For other measurements, one independent sample was taken from each box as a biological replicate. There were three replicates per treatment.

## Results

### SL biosynthesis is induced by *M. incognita*

**Fig. 1.**
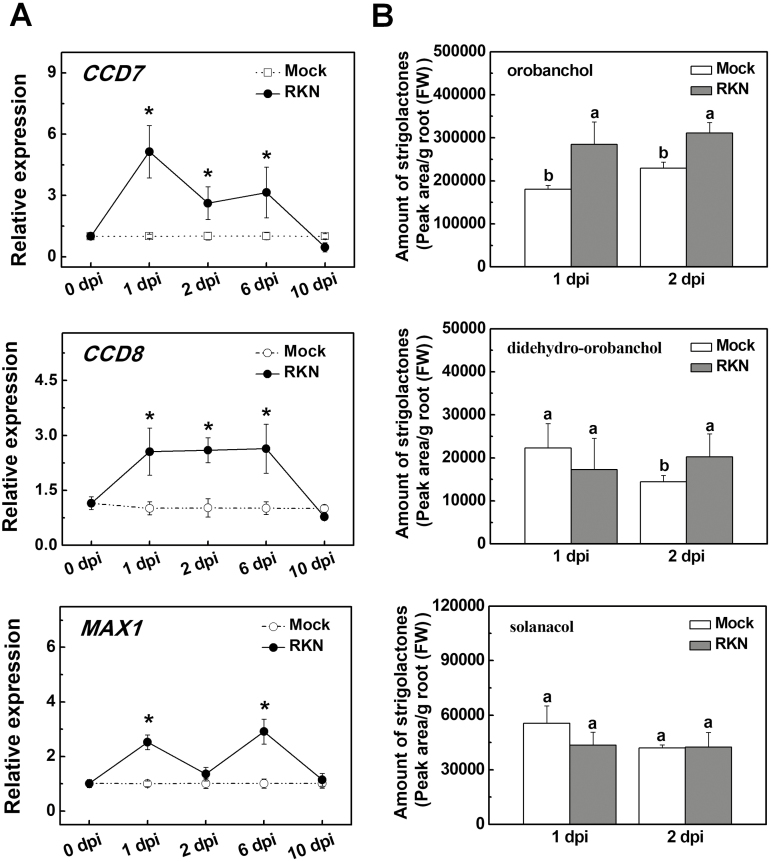
Infection with root-knot nematodes (RKNs) induces biosynthesis of strigolactones (SLs) in tomato roots. (A) Time-course of the relative expression of genes involved in SL biosynthesis in the roots. Values are expressed relative to the *Actin* gene. Significant differences compared with values before RKN inoculation were determined using Student’s *t*-test: *P<0.05. (B) Accumulation of SLs in the roots as determined by UPLC-MS/MS. Different letters indicate significant differences compared with the mock as determined by ANOVA followed by Duncan’s multiple range test (*P*<0.05). Data are means (±SD) of three replicates.

To determine whether SL biosynthesis is involved in the defense against RKNs (*M. incognita*) in tomato, we first examined the time-course of gene transcripts involved in SL biosynthesis (*CCD7*, *CCD8*, and *MAX1*) in response to RKN infection in a susceptible genotype (cultivar Ailsa Craig). As shown in Fig. 1A, RKN infection significantly induced the transcription of these genes in the roots by ~2–5-fold at 1 d post-infection (dpi), but the transcript levels decreased to values close to those of the control at 10 dpi. UPLC-MS/MS analysis indicated that RKN infection induced the accumulation of orobanchol and didehydro-orobanchol but did not alter the accumulation of solanacol in the roots at 1 dpi or 2 dpi (Fig. 1B). The transcripts of *PLANT DEFENSE FACTOR* (*PDF*), *PROTEINASE INHIBITOR1* (*PI-1*), and *PI-2*, which are involved in the defense response, were up-regulated whereas that of *MYC2* was down-regulated at 3 h after the RKN infection (Supplementary Fig. S1). These results suggested that SL biosynthesis was induced in response to RKN infection and this increase was associated with the induction of the defense response.

### SLs play a positive role in defense against RKNs

As SL biosynthesis was induced after RKN infection in the roots, we then investigated whether this was linked to defense against RKNs. To this end, we used a VIGS approach to construct tomato plants with silencing of genes related to SL biosynthesis, namely pTRV-*CCD7*, pTRV-*CCD8*, and pTRV-*MAX1*. Compared with the empty-vector plants (pTRV), these VIGS plants exhibited reduced transcription of their corresponding genes by ~77–80% (Supplementary Fig. S2A). In addition, the root extracts of the VIGS plants contained lower contents of orobanchol, solanacol, and didehydro-orobanchol, and were less efficient at stimulating the germination of *P. aegyptiaca* seeds compared with the pTRV plants (Supplementary Fig. S2B). These results suggested that SL biosynthesis was significantly suppressed in the roots of the VIGS plants. Consistent with the role of SLs in plant development (Kohlen *et al.*, 2012), VIGS plants displayed increased shoot branching, reduced plant height, and increased mass of roots in the absence of RKN infection (Supplementary Table S4).

**Fig. 2.**
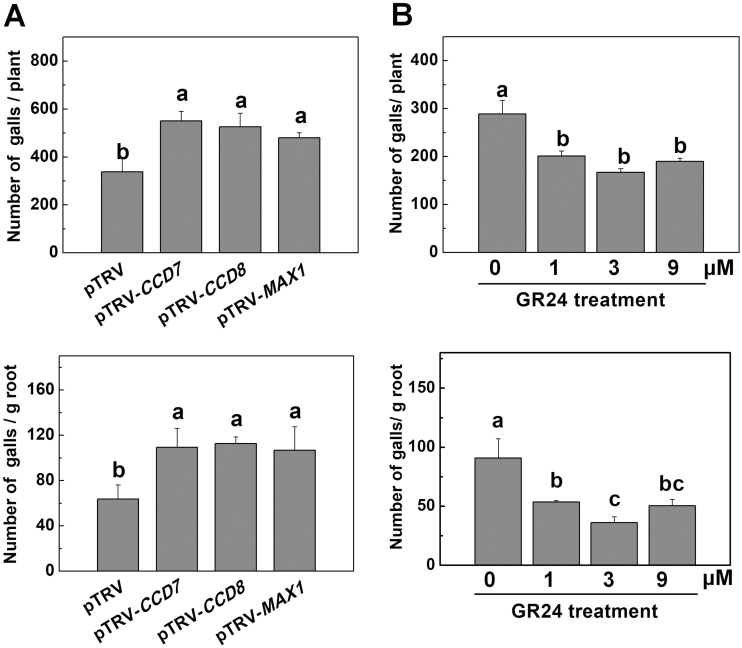
Influence of strigolactone (SL) biosynthesis and application of GR24 on the susceptibility of tomato plants to root-knot nematodes (RKNs). (A) The number of RKN galls in *CCD7*-, *CCD8*-, and *MAX1*-silenced plants. Empty pTRV vectors served as controls. (B) The number of RKN galls in wild-type plants (cv. Ailsa Craig) when roots were drenched with different concentrations of the synthetic SL analog GR24 (1–9 μM). Distilled water solution containing the equivalent concentration of solvent served as the control (0 μM). The GR24 treatment was applied 24 h before RKN infection. Gall numbers were determined 4 weeks after RKN infection. Thirty plants per treatment were used in each experiment. The RKN experiment was repeated three times with similar results each time, and data from one representative experiment are presented. Data are means (±SD) of three replicates. Different letters indicate significant differences as determined by ANOVA followed by Duncan’s multiple range test (*P*<0.05).

We then examined the role of SL biosynthesis in the defense response against inoculation with 1000 *M. incognita* J2s per plant. After 4 weeks, roots of the VIGS plants suffered more severe RKN infection, with more females in roots and larger galls relative to the pTRV plants (Supplementary Fig. S3A, B). In addition, gall numbers in the pTRV-*CCD7*, pTRV-*CCD8*, and pTRV-*MAX1* plants increased by 62.9%, 55.6%, and 42.2%, respectively, relative to that in pTRV plants (Fig. 2A). Similarly, the gall number per unit weight of root tissue in the pTRV-*CCD7*, pTRV-*CCD8*, and pTRV-*MAX1* plants increased by 71.6%, 76.9%, and 67.6%, respectively (Fig. 2A). Although VIGS plants showed increased root mass relative to the pTRV plants in the absence of RKNs (Supplementary Table S4), this difference was not observed in the presence of RKNs (Supplementary Fig. S3C).

Next, we examined the effect of root application of *rac*GR24, a synthetic racemic mixture of SLs analogs, on defense against RKNs in wild-type tomato plants (cv. Alisa Craig). Drenching roots with *rac*GR24 solution had a negative effect on the transcription of the SL biosynthesis genes *CCD7* and *CCD8* in the roots (Supplementary Fig. S4A), as has been previously observed in Arabidopsis (Mashiguchi *et al.*, 2009). Both extracts from *rac*GR24-treated roots and *rac*GR24 solution could promote the germination of *P. aegyptiaca* seeds more efficiently than the respective controls (Supplementary Fig. S4B). In addition, *rac*GR24 did not affect the hatching of RKN eggs at the concentration used (Supplementary Fig. S4C), excluding the possibility of a direct harmful effect of *rac*GR24 on the RKN. As shown in Fig. 2B and Supplementary Fig. S5, root applications of *rac*GR24 at different concentrations (1–9 μM) significantly decreased the number of galls per plant, the number of galls per unit weight of root tissue, and the number of females. At the same time, root fresh weight was increased by 18.8%, 43.8%, and 18.8% after application of *rac*GR24 at 1 μM, 3 μM, and 9 μM, respectively (Supplementary Fig. S5C). Taken together, our results indicated that SL biosynthesis plays an important role in the defense against RKNs in tomato plants.

**Fig. 3.**
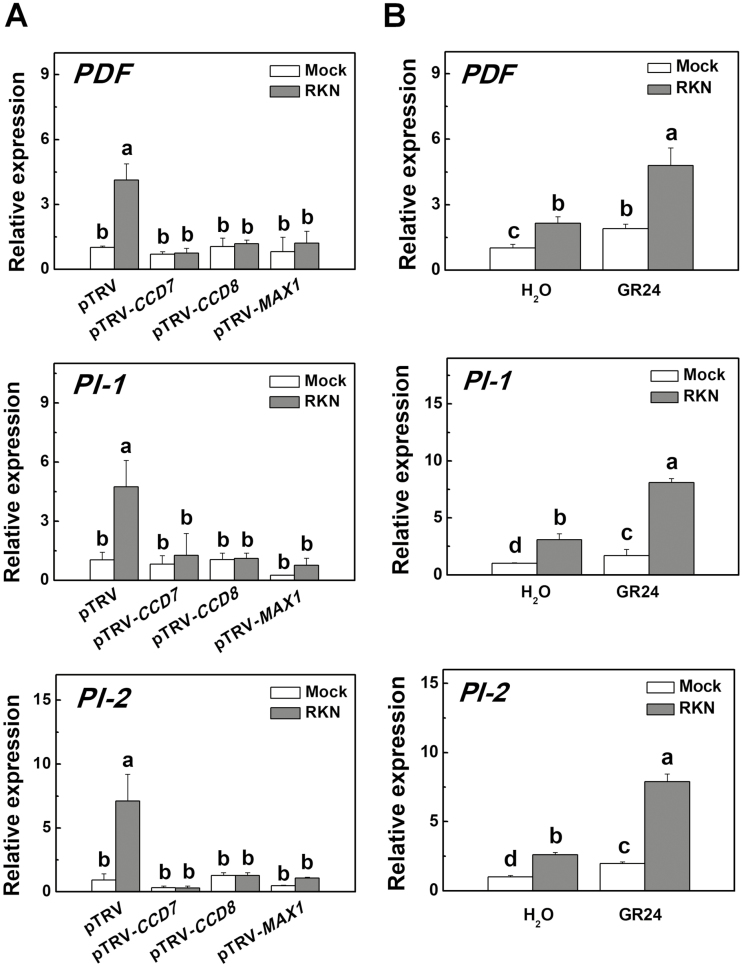
Transcription of defense-related genes in tomato plants with silencing of strigolactone (SL)-related genes and in wild-type plants drenched with GR24 in response to infection with root-knot nematodes (RKNs). (A) The relative expression of *PDF*, *PI-1*, and *PI-2* in *CCD7*-, *CCD8*-, and *MAX1*-silenced plants. Empty pTRV vectors served as controls. Values are expressed relative to the *Actin* gene. (B) The relative expression of *PDF*, *PI-1*, and *PI-2* in wild-type plants (cv. Ailsa Craig) when roots were drenched with GR24 (3 μM solution). Distilled water solution containing the equivalent concentration of solvent served as the control. RNA was isolated from root samples collected 24 h after RKN infection, and transcript levels were determined using qRT-PCR. Data are means (±SD) of three replicates. Different letters indicate significant differences as determined by ANOVA followed by Duncan’s multiple range test (*P*<0.05).

To determine whether the SL-induced defense response against RKNs was linked to the up-regulation of defense-related genes, we analysed the transcripts of *PDF*, *PI-1*, and *PI-2* in the VIGS plants, and also wild-type plants pretreated with *rac*GR24 in the presence or absence of RKN infection. The results showed that silencing of *CCD7*, *CCD8*, or *MAX1* did not alter the transcription of *PDF*, *PI-1*, or *PI-2* in the absence of RKN infection (Fig. 3A). RKN infection significantly induced the transcription of *PDF*, *PI-1*, and *PI-2* in the pTRV plants but it had no effect in the VIGS plants. Application of *rac*GR24 to wild-type plants markedly induced the transcription of *PDF*, *PI-1*, and *PI-2*, especially in the presence of RKN (Fig. 3B). We then co-silenced the *PI-1* and *PI-2* genes in the plants (pTRV-*PI-1/2*) and found that they exhibited more RKN galls than the pTRV plants (Supplementary Fig. S6A, B). Importantly, silencing both the *PI-1* and *PI-2* genes compromised *rac*GR24-induced defense (Fig. S6B, C). Taken together, these results suggested that SL biosynthesis is linked to the defense response against RKNs in tomato plants.

### Crosstalk between SLs and other hormones in response to nematode attack

**Fig. 4.**
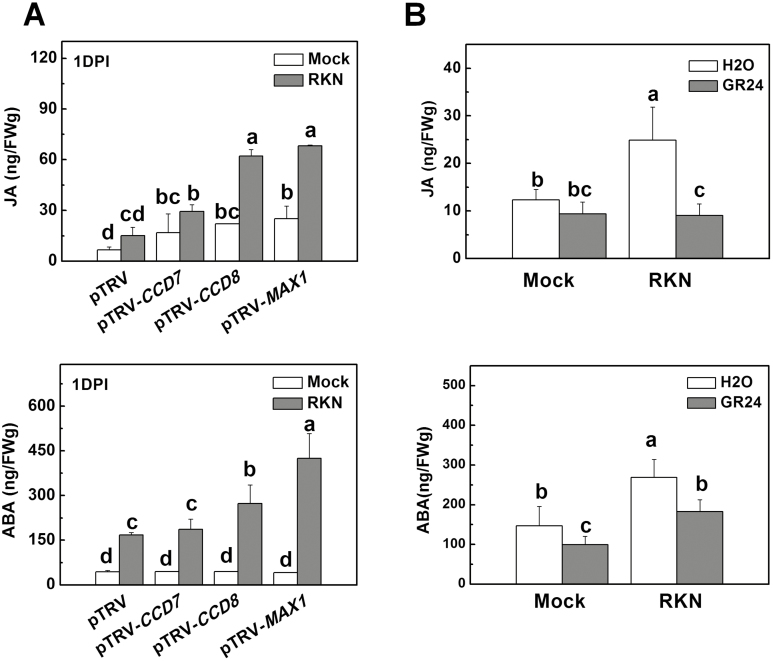
Endogenous jasmonic acid (JA) and abscisic acid (ABA) contents in the roots of tomato plants with silencing of strigolactone (SL-)related genes and in wild-type plants drenched with GR24 in response to infection with root-knot nematodes (RKNs). (A) Endogenous JA and ABA contents in the roots of *CCD7-*, *CCD8*-, and *MAX1*-silenced plants. Empty pTRV vectors served as controls. (B) Endogenous JA and ABA contents in the roots of wild-type plants (cv. Ailsa Craig) when roots were drenched with GR24 (3 μM solution) 24 h before RKN infection. Distilled water containing the equivalent concentration of solvent served as the control. Root samples were collected 24 h after RKN infection. The JA and ABA contents were determined using UPLC-MS/MS. Data are means (±SD) of three replicates. Different letters indicate significant differences as determined by ANOVA followed by Duncan’s multiple range test (*P*<0.05).

**Fig. 5.**
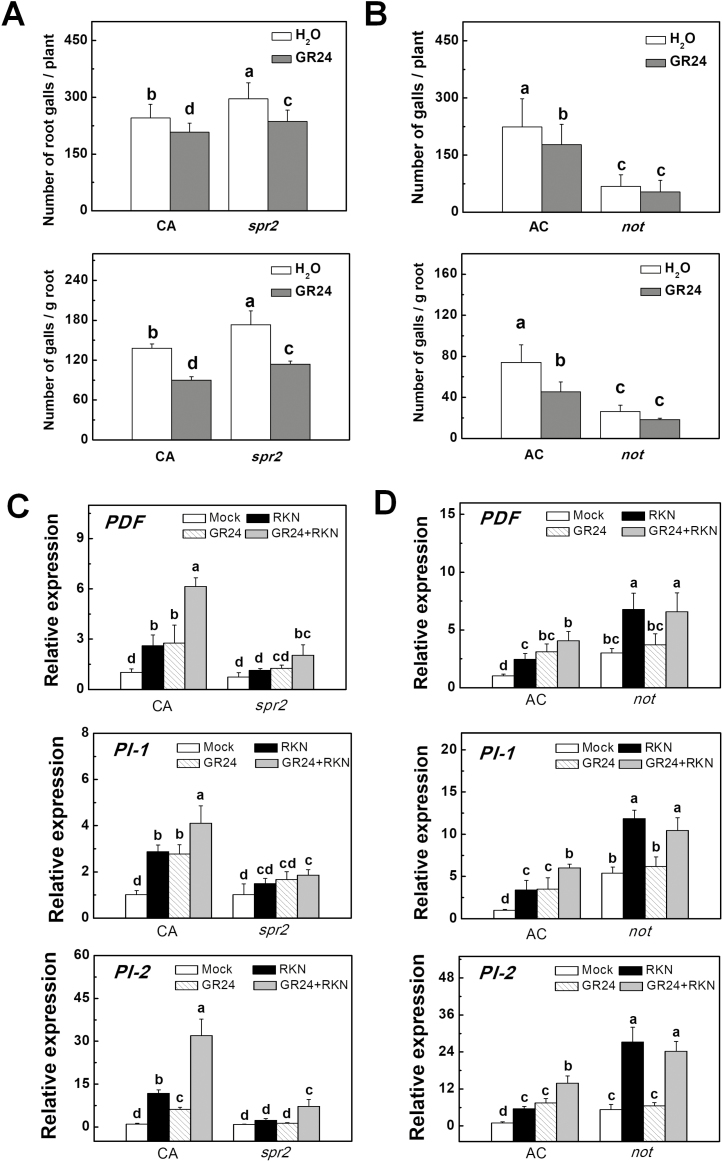
Effects of application of GR24 on the defense response in jasmonic acid (JA) and abscisic acid (ABA) mutants and wild-type tomato plants in response to infection with root-knot nematodes (RKNs). (A) The number of RKN galls in the JA biosynthesis mutant *spr2* and its background wild-type cv. Castlemart (CA) in the presence or absence of GR24 drenching. (B) The number of RKN galls in the ABA biosynthesis mutant *notabilis* (*not*) and its background wild-type cv. Ailsa Craig (AC) in the presence or absence of GR24 drenching. The concentration of the GR24 solution was 3 μM, and the treatment was applied 24 h before RKN infection. Gall numbers were determined 4 weeks after RKN infection. (C) The relative expression of *PDF*, *PI-1*, and *PI-2* in *spr2* and CA in the presence or absence of GR24. (D) The relative expression of *PDF*, *PI-1*, and *PI-2* in *not* and AC in the presence or absence of GR24. RNA was isolated from root samples collected 24 h after RKN infection, and transcript levels were determined using qRT-PCR. Values are expressed relative to the *Actin* gene. Data are means (±SD) of three replicates. Different letters indicate significant differences as determined by ANOVA followed by Duncan’s multiple range test (*P*<0.05).

Plant hormones such as JA and ABA are known for their positive and negative roles in defense responses against nematodes (Nahar *et al.*, 2011, 2012). To determine whether SL-induced defense against RKNs in tomato was caused by altered hormone homeostasis in the roots, we compared changes in the levels of JA and ABA in the roots of pTRV-*CCD7*, pTRV-*CCD8*, and pTRV-*MAX1* VIGS plants, and also in wild-type plants that had been pretreated with *rac*GR24 one day before RKN infection. After 24 h of RKN infection, there was greater accumulation of JA and ABA in the roots of the VIGS plants than in the pTRV plants (Fig. 4A). RKN-induced accumulation of JA and ABA was reduced in wild-type plants pretreated with *rac*GR24 (Fig. 4B). These results indicated that SL biosynthesis affected the accumulation of JA and ABA during RKN infection. Next, we examined whether SLs induced defense against RKNs by altering JA and ABA signaling. To this end, mutants deficient in the biosynthesis of JA (*spr2*) and ABA (*not*) were used. Compared with their respective background wild-types (WTs; Castlemart or Ailsa Craig), *spr2* plants had more galls in the roots, whereas *not* plants had fewer galls (Fig. 5A, B). Application of *rac*GR24 to the roots reduced the number of galls in the WT plants of both cultivars and also in the *spr2* plants, and resulted in an increase in the fresh weight of the roots (Supplementary Fig. S7). In contrast, *rac*GR24 had little effect on gall development in the *not* plants and no significant effect on root weight was observed (Fig. 5B, Supplementary Fig. S7). RKN-induced transcription of *PDF*, *PI-1*, and *PI-2* was reduced in *spr2* plants but it was promoted in *not* plants, and *rac*GR24 had no significant effect on the transcripts of the genes in *not* plants (Fig. 5C, D). Although the transcription of *PDF*, *PI-1*, and *PI-2* correlated well with RKN resistance in SL-deficient and *rac*GR24-treated plants, *rac*GR24 induced resistance to RKNs in *spr2* plants, which showed compromised induction of *PDF*, *PI-1*, and *PI-2*. Furthermore, *rac*GR24 and RKNs showed additive effects on the induction of these defense genes (Fig. 5C). It is likely that these defense-related genes are regulated by multiple pathways other than SLs (such as JA signaling) in response to RKNs. On the basis of our results, we speculated that SLs may be able to induce defense responses against RKNs through mechanisms other than the JA-dependent induction of *PDF*, *PI-1*, and *PI-2*, in which the ABA pathway plays a significant role.

### SL induces defense against RKNs by suppressing the transcription of *MYC2*

**Fig. 6.**
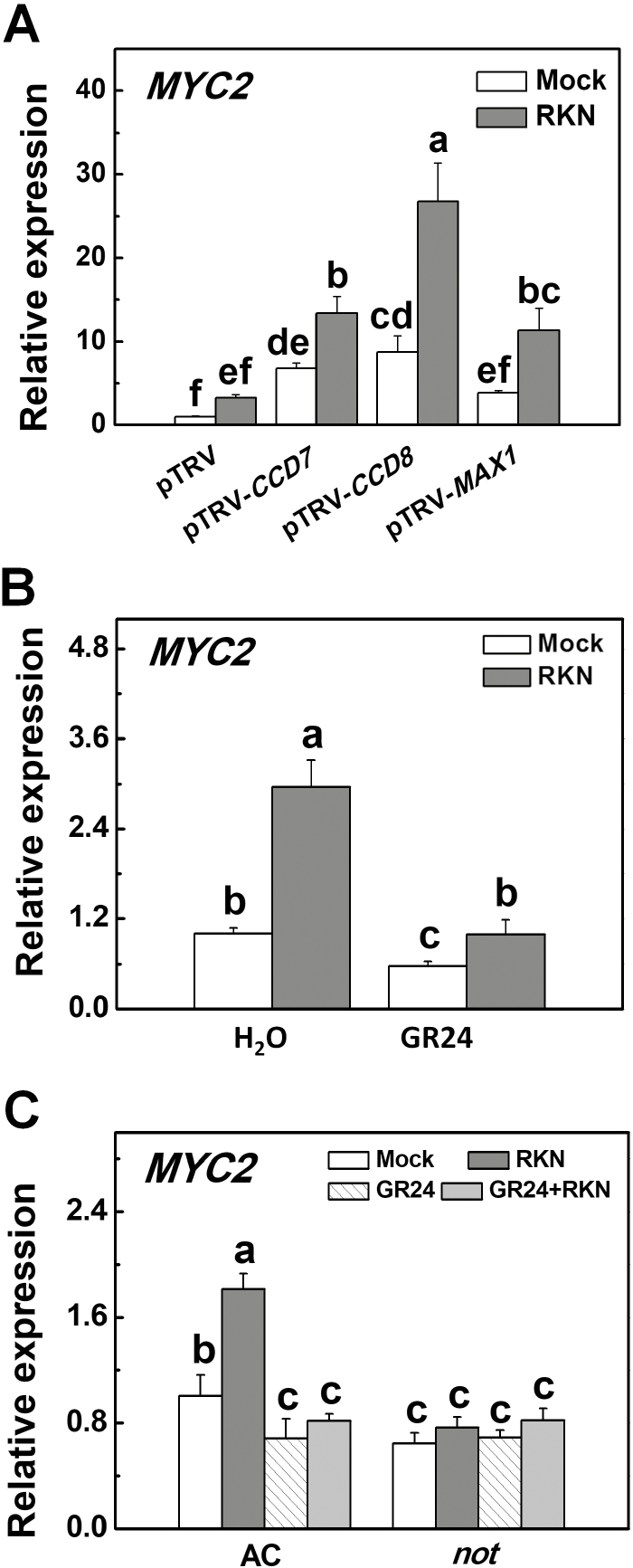
Transcription of *MYC2* in the roots of tomato plants with silencing of strigolactone (SL-)related genes and in an abscisic acid (ABA) biosynthesis mutant in the presence or absence of GR24 treatment in response to infection with root-knot nematodes (RKNs). (A) The relative expression of *MYC2* in *CCD7*-, *CCD8*-, and *MAX1*-silenced plants. Empty pTRV vectors served as controls. (B) The relative expression of *MYC2* in wild-type cv. Ailsa Craig plants (AC) when roots were drenched with a solution of GR24 at 3 μM. Distilled water containing the equivalent concentration of solvent served as the control. (C) The relative expression of *MYC2* in the ABA biosynthesis mutant *not* and its background wild-type cv. Ailsa Craig (AC) plants in the presence or absence of GR24 drenching. GR24 was applied at a concentration of 3 μM 24 h before RKN infection. RNA was isolated from root samples collected 24 h after RKN infection, and transcript levels were determined using qRT-PCR. Values are expressed relative to the *Actin* gene. Data are means (±SD) of three replicates. Different letters indicate significant differences as determined by ANOVA followed by Duncan’s multiple range test (*P*<0.05).

MYC2 mediates crosstalk between ABA and JA in plant stress responses, but its role in defense against RKNs is unknown (Anderson *et al.*, 2004; Lorenzo *et al.*, 2004). We found that RKN infection induced the transcription of *MYC2* in roots at 1 dpi (Fig. 6A–C). Importantly, *CCD7*-, *CCD8*-, and *MAX1*-silenced plants displayed increased transcription of *MYC2* in the roots, and this increase was especially significant in the presence of RKN (Fig. 6A). In agreement with this, application of *rac*GR24 to the roots down-regulated the transcription of *MYC2* in the roots regardless of RKN infection (Fig. 6B). We also examined whether SL-induced defense against RKNs was attributable to ABA-induced changes in the transcription of *MYC2* in the roots. After RKN inoculation, *not* plants displayed decreased transcription of *MYC2* in the roots as compared to WT plants (Fig. 6C). Moreover, *rac*GR24 suppressed the induction of *MYC2* by RKN infection in WT plants but had no effect in the *not* plants. These results indicated that SLs decreased *MYC2* transcription by altering the ABA pathway.

**Fig. 7.**
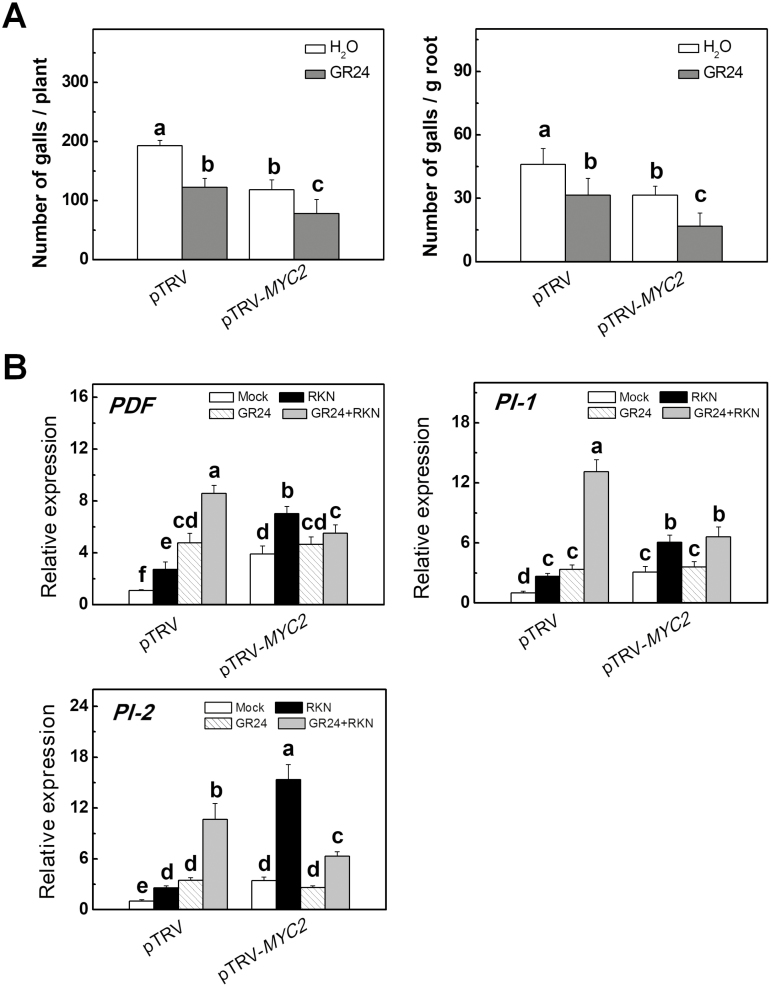
*MYC2*-silenced tomato plants showed decreased susceptibility to infection by root-knot nematodes (RKNs). (A) The number of RKN-induced root galls in *MYC2*-silenced plants in the presence or absence of GR24. Empty pTRV vectors served as controls. Gall numbers were determined 4 weeks after RKN infection. Thirty plants per treatment were used in each experiment. (B) The relative expression of *PDF*, *PI-1*, and *PI-2* in *MYC2*-silenced plants in the presence or absence of GR24. Empty pTRV vectors served as controls. GR24 was applied at a concentration of 3 μM 24 h before RKN infection. RNA was isolated from root samples collected 24 h after RKN infection, and transcript levels were determined using qRT-PCR. Values are expressed relative to the *Actin* gene. Data are the means of three replicates (±SD). Different letters indicate significant differences as determined by ANOVA followed by Duncan’s multiple range test (*P*<0.05).

To further determine the role of *MYC2* in SL-induced defense against RKNs in tomato, we used a VIGS approach to silence *MYC2* (pTRV-*MYC2*) and, after confirmation of effective silencing (Supplementary Fig. S8), inoculated the plants with RKNs. pTRV-*MYC2* plants showed increased defense against RKNs, as indicated by a 31.5% decrease in the number of galls per unit weight of root tissue and a 38.2% decrease in the number of galls per plant (Fig. 7A). Application of *rac*GR24 to the roots increased the defense against RKNs in both the pTRV and pTRV*-MYC2* plants. qRT-PCR analysis revealed that the transcription of *PDF*, *PI-1*, and *PI-2* in the roots was up-regulated in the pTRV-*MYC2* plants, especially after RKN infection (Fig. 7B). Silencing of *MYC2* abolished *rac*GR24-induced transcription of *PDF*, *PI-1*, and *PI-2* (Fig. 7B). These results suggested that SLs regulate *MYC2* transcription in an ABA-dependent manner and that this regulatory mechanism is critical for the SL-induced defense against RKNs.

## Discussion

**Fig. 8.**
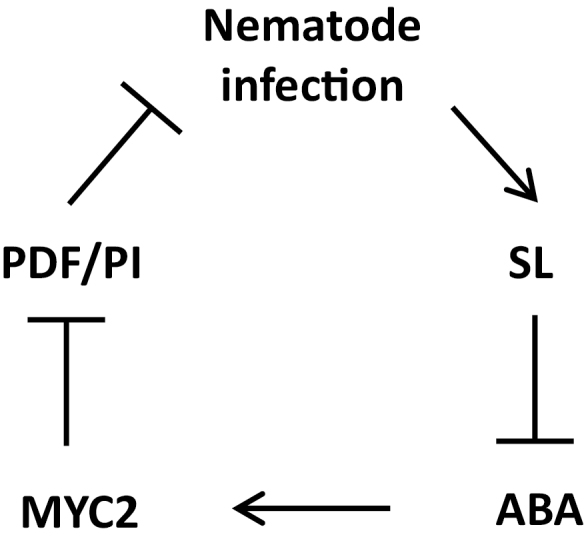
A model describing the mechanisms by which strigolactone (SL) regulates the tomato defense response against infection by root-knot nematodes (RKNs) through crosstalk with the abscisic acid (ABA) pathway. Arrows indicate stimulation, blocked lines indicate suppression.

Strigolactones (SLs) influence different processes in plants, including shoot branching, root development, leaf senescence, and responses to environmental stresses such as nutrient limitation, drought, and salinity (Kapulnik *et al.*, 2011*b*; de Jong *et al.*, 2014; Ha *et al.*, 2014; Ueda and Kusaba, 2015). However, the role of SLs in biotic stress responses is not well established. Here, we present evidence showing that SLs are critical for defense against root-knot nematodes (RKNs) in tomato plants. JA and ABA, which are positive and negative regulator of RKN resistance, were both suppressed by SL in RKN-infected roots. SL-mediated RKN resistance was partially independent of JA signaling, but can be attributed to suppression of ABA-dependent regulation of *MYC2*, which functions as a negative regulator of defense against RKNs (Fig. 8).

### SLs positively regulate defense against RKNs in tomato

Previous reports have indicated that SLs played positive roles in defense against fungal pathogens such as *B. cinerea* and *A. alternata* in tomato (Torres-Vera *et al.*, 2014). The Arabidopsis SL-insensitive mutant *max2* is susceptible to the bacterial pathogens *Pseudomonas syringae* and *Pectobacterium carotovorum* (Piisilä *et al.*, 2015). Similarly, Arabidopsis *max2* and the SL-related mutants *max1*, *max3*, and *max4* are hypersensitive to the actinomycetous pathogen *Rhodococcus fascians* that causes leaf gall syndrome (Stes *et al.*, 2015). However, SLs do not appear to influence susceptibility to infection by the necrotrophic soil-borne oomycete *Pythium irregulare* or the hemibiotrophic fungus *Fusarium oxysporum* in pea (*Pisum sativum*) (Steinkellner *et al.*, 2007; Dor *et al.*, 2011; Blake *et al.*, 2016; Foo *et al.*, 2016). Here, we provide multiple lines of evidence indicating that SLs positively regulate the defense against RKNs in tomato plants. First, transcripts of the SL biosynthetic genes *CCD7*, *CCD8*, and *MAX1* increased in roots in response to RKN infection and this was accompanied by accumulation of endogenous orobanchol and didehydro-orobanchol (Fig. 1). Second, silencing of *CCD7*, *CCD8*, or *MAX1* increased plant susceptibility to RKNs, as shown by more nematode galls in the roots as compared to control plants (Fig. 2A). Although *CCD7*-, *CCD8*-, and *MAX1*-silenced plants had more developed root systems in the absence of RKN infection (Supplementary Fig. S3A), they had more galls per plant and per unit weight of root tissues when they were infected. It therefore appeared that the increase in RKN galls was not due to the changes in root mass. Third, application of the SL analog *rac*GR24 to the roots significantly reduced the number of galls (Fig. 2B). These results strongly suggested that both SL biosynthesis and signaling are important in defense against RKNs in tomato. However, the use of *rac*GR24 can induce both the SL and karrikin pathways (Scaffidi *et al.*, 2014), and hence an effect of the latter cannot be excluded.

Suppressed expression of *PI-2* has been observed in *CCD8*-RNAi plants (Torres-Vera *et al.*, 2014). Our present study demonstrated that SLs were involved in the regulation of genes related to defense against RKN. RKN infection induced the transcription of *PDF*, *PI-1* and *PI-2*; this induction was significantly reduced in the *CCD7*-, *CCD8*-, and *MAX1*-silenced plants but was promoted by the exogenous application of *rac*GR24 in non-silenced wild-type (WT) plants (Fig. 3). Proteinase inhibitors (PIs) play a positive role in defense against nematodes (Koiwa *et al.*, 1997; Ali *et al.*, 2017). Consistent with previous studies, we found that co-silencing of *PI-1* and *PI-2* (pTRV*-PI-1/2*) reduced defense against RKNs, and exogenous application of *rac*GR24 did not enhance defense in pTRV*-PI-1/2* plants (Supplementary Fig. S4). These results suggested that SLs might regulate nematode defense at the transcription level and in a manner that is dependent on *PI-1/2*.

### SL-induced defense against nematodes is linked to changes in hormone homeostasis in roots

JA is known to be a positive regulator of defense against nematodes (Cooper *et al.*, 2005; Fujimoto *et al.*, 2011; Nahar *et al.*, 2011). Foliar application of JA has been shown to induce a strong systemic defense response in roots, thus reducing nematode reproduction in the plants (Nahar *et al.*, 2011). Here, we observed that the roots of *CCD7*, *CCD8*-, and *MAX1*-silenced plants exhibited increased accumulation of JA as compared to control plants (Fig. 4). However, the higher JA levels were not associated with enhanced defense against RKNs in the silenced plants (Fig. 2). Exogenous application of *rac*GR24 decreased the JA levels in the roots of WT plants and effectively enhanced the defense against RKNs in the JA biosynthesis mutant *spr2*, which showed reduced defense against RKNs compared to the WT (Figs 4, 5). Therefore, it is unlikely that SLs induced defense by increasing the biosynthesis of JA; rather, activation by SLs of a JA-independent defense pathway against RKNs may compensate for the decrease in JA levels.

ABA has been shown to be a negative regulator of defense against nematodes in rice and tomato (Nahar *et al.*, 2012; Song *et al.*, 2018) with foliar ABA treatment suppressing rice basal immunity against nematodes, whereas inhibition of ABA biosynthesis leads to a substantial reduction in disease severity. There is evidence that ABA-induced susceptibility to nematodes in rice involves the repression of the JA pathway (Nahar *et al.*, 2012). Here, we found that the ABA biosynthesis mutant *not* showed stronger defense against RKNs than WT plants (Fig. 5), indicating that the negative role of ABA in nematode resistance is conserved among different plant species. In addition, we found that the compromised defense in plants silenced for SL biosynthesis genes was associated with increased accumulation of both ABA and JA in roots after RKN infection (Figs 2, 4). Moreover, application of *rac*GR24 enhanced defense but reduced ABA accumulation in response to RKN infection. However, application of *rac*GR24 was not able to effectively enhance defense in the *not* mutant (Fig. 5). SLs and ABA show extensive crosstalk in the regulation of plant growth and development. SLs promote seed germination through modulating ABA levels by up-regulating ABA catabolic genes (Lechat *et al.*, 2012; Toh *et al.*, 2012). In addition, the expression of ABA biosynthesis, catabolism, transport, and signaling genes is altered in SL-insensitive mutants during the regulation of seedling growth and drought tolerance (Mashiguchi *et al.*, 2009; Bu *et al.*, 2014; Li *et al.*, 2017). Based on these results, we speculate that SLs induce defense against RKNs by inhibiting the accumulation of ABA through regulation of either its biosynthesis or catabolism. Notably, ABA biosynthesis mutants show a decrease in SL accumulation (López-Ráez *et al.*, 2010). Therefore, ABA seems to act downstream of SLs to regulate the defense response to RKNs.

In Arabidopsis, both SL-deficient and SL-insensitive mutants exhibit weak tolerance to drought and salt stress coupled with decreased ABA responsiveness (Bu *et al.*, 2014; Ha *et al.*, 2014). In contrast to the changes of ABA content in the roots, we observed a ~22.3–32.4% decrease in the accumulation of ABA in the leaves of *CCD7*-, *CCD8*-, and *MAX1*-silenced plants under optimal growth conditions (data not shown). *CCD8*-RNAi tomato plants have decreased ABA content in the leaves (Torres-Vera *et al.*, 2014). It is likely that the effects of SLs on ABA biosynthesis are organ-specific. Collectively, these results suggest an intricate crosstalk between SLs and ABA in the stress response.

### MYC2 participates in SL-induced defense against nematodes

We found that MYC2 participated in SL-induced defense against nematodes by functioning as a negative regulator in tomato plants. Silencing of *CCD7*, *CCD8*, or *MAX1* resulted in increased transcription of *MYC2* and accumulation of ABA but reduced defense against RKNs (Figs 2, 6A). In contrast, application of *rac*GR24 inhibited the expression of *MYC2*, with a decrease in ABA accumulation followed by improved defense against RKNs. Furthermore, transcription levels of *MYC2* in resistant *not* mutants were strongly reduced compared to the WT (Fig. 6C). It was notable that silencing of *MYC2* significantly enhanced defense against RKNs (Fig. 7). Collectively, our results suggest that MYC2 plays a crucial role in SL-induced defense against nematodes in an ABA-dependent manner.

In Arabidopsis, AtMYC2 functions as both a positive and negative regulator of JA-responsive genes in JA signaling (Lorenzo *et al.*, 2004). *myc2* mutant plants display compromised JA-induced defense to the herbivore *Helicoverpa armigera* and show increased susceptibility to the herbivore *Spodoptera littoralis* (Dombrecht *et al.*, 2007; Fernández-Calvo *et al.*, 2011). In addition, *myc2* mutants are unable to mount rhizobacteria-induced systemic defense against *P. syringae* and *Hyaloperonospora parasitica* (Pozo *et al.*, 2008). In tomato, the MYC2 homolog acts downstream of the JA receptor to orchestrate JA-mediated activation of both the wounding and pathogen responses (Du *et al.*, 2017). However, the JA-deficient *spr2* mutant and *MYC2*-silenced plants showed opposite phenotypes with regards to defense against nematodes (Figs 5, 7). It is therefore unlikely that MYC2 is involved in JA-induced defense against nematodes in tomato.

Interestingly, MYC2 has been shown to be a positive regulator of ABA signaling. *MYC2* is an ABA-responsive gene and *myc2* mutants show reduced ABA sensitivity (Abe *et al.*, 2003; Lorenzo *et al.*, 2004). Furthermore, MYC2 is capable of activating the expression of the ABA-response genes (Abe *et al.*, 2003). Consistent with these results, the induction of *MYC2* by RKN infection was abolished in the ABA-deficient *not* mutant (Fig. 6). In addition, *rac*GR24 suppressed the transcription of *MYC2* in WT plants but not in *not* mutant plants (Fig. 6). Therefore, ABA plays a crucial role in the regulation of *MYC2*. SLs may thus enhance defense against nematodes by inhibiting the expression of *MYC2* through regulation of ABA levels. However, silencing of *MYC2* did not completely abolish *rac*GR24-induced defense against nematodes, but it reduced *rac*GR24-induced transcription of *PDF*, *PI-1*, and *PI2* (Fig. 7). These results suggest that other MYC2-independent signaling pathways are involved in SL-induced defense against RKNs, whilst MYC2 is important for the regulation of transcription of *PDF*, *PI-1*, and *PI2*.

### Conclusions

Overall, our results demonstrate that SLs function as a positive regulator in the defense against nematode attack. ABA appears to act downstream of SL in the defense response to RKNs by suppressing the expression of *MYC2*, which negatively regulates defense, whereas *PDF* and *PI* play major roles in the SL-mediated defense response. Our results not only highlight the importance of SLs in biotic responses but also identify novel targets for the genetic improvement of defense in tomato.

## Supplementary data

Supplementary data are available at *JXB* online.

Fig. S1. Time-course of expression of defense-related genes in tomato roots in response to RKN infection.

Fig. S2. Silencing efficiency of SL biosynthesis genes in wild-type tomato roots.

Fig. S3. Influence of silencing of *CCD7*, *CCD8*, and *MAX1* on RKN development and root weight of tomato plants.

Fig. S4. Effect of GR24 application on the expression of SLs biosynthesis genes and RKN growth.

Fig. S5. Influence of GR24 on RKN development and root weight in tomato plants.

Fig. S6. Effects of GR24 application on defense against RKN of *PI-1/2* co-silenced plants.

Fig. S7. Root weights in wild-type, *spr2*, and *not* plants after RKN infection.

Fig. S8. Silencing efficiency of *MYC2* in wild-type tomato roots.

Table S1. PCR primers and restriction sites for VIGS vector construction.

Table S2. Parameters used to detect phytohormones and related compounds through UPLC-MS/MS.

Table S3. Primers used for qRT-PCR assays.

Table S4. Effects of VIGS on plant growth parameters.

Supplementary Tables S1-S4

Supplementary Figures S1-S8
