# Supplementary material for: Strigolactones positively regulate defense against root-knot nematodes in tomato
Source: J Exp Bot. 2018 Dec 21;70(4):1325–37. doi: 10.1093/jxb/ery439 (PMC6382333; doi:10.1093/jxb/ery439)
Supplement: Supplementary Tables S1-S4 [file ery439_suppl_supplementary_tables_s1-s4.pdf]

Supplemental Table 1. PCR primers and restriction sites for VIGS vectors construction.

Gene	Restriction sites	Forward primer	Reverse primer
CCD7	EcoRI BamHI	5'-CGgaattcCTAAAACAAAAGCCAAAATG-3'	5'-GCggatc**c**ATCCCAATAGGCAGTAAC-3'
CCD8	EcoRI BamHI	5'-CGgaattcTCTTCACATCTATGGCTTCT-3'	5'-GCggatccATCTGGTGGTGGAACTATTA-3'
MAX1	EcoRI BamHI	5'-CGgaattcGATTTGATCTCCTCCTTC-3'	5'-GCggatccGCATCAGCAACAATAACT-3'
PI-1	EcoRI BamHI	5’-CGgaattcGGAAAATCCATCCATAAC-3’	5’-GCggatccTGAACATACATGAACCCT-3’
PI-2	EcoRI BamHI	5’-CGgaattcGTGGTCATCTTGGGTTTG-3’	5’-GCggatccCCAGTGGGATAAATAAACG-3’
MYC2	XbaI BamHI	5'-CGtctagaTGCTGCTGTGGTTGGT-3'	5'-GCggatccTCCGATGCTCCTGCTC-3'

Supplemental Table 2. Parameters used for detection of phytohormones and related compounds by HPLC-MS/MS.

**Compound**	**Capillary CID[1] (V)**	**Molecular ion [M-H] (*m/z*)**	**Fragment ion (*m/z*)**	**CE[2] (V)**	**Reference**
JA	50	209.1	59.1	2	Wu *et al*. (2007)
D_5-JA (IS)	116	214.3	62.1	8	Alba *et al*. (2015)
ABA	75	263.1	153	0	Durgbanshi *et al*. (2005)
D_6-ABA (IS)	162	269.3	159.2	0	Durgbanshi *et al*. (2005)

[1]collision-induced dissociation; [2]collision energy; IS, internal standard.

Supplemental Table 3. Primers used for qRT-PCR assays.

Gene	Accession numbers	Forward primer	Reverse primer
Actin	Solyc03g078400	5'-TGGTCGGAATGGGACAGAAG-3'	5'-CTCAGTCAGGAGAACAGGGT-3'
CCD7	Solyc01g090660	5'-AGCAGATTTTCCAGCGATGAA-3'	5'-AAAGGAAAATGGGGTAGCGC-3'
CCD8	Solyc08g066650	5'-CCTTCAACGGCAAGGATGTC-3'	5'-AAGGCCTCTTAGCACCACAA-3'
MAX1	Solyc08g062950	5'-GGTGTTACATTGTCTCGCCC-3'	5'-CTCCAAGAGCCAACCAAACC-3'
MYC2	Solyc08g076930	5'-TAGCCACACTGGAGGCAAGATT-3'	5'-CTAGGTCTAATTCCATGAGCGC-3'
PDF	Solyc07g006380	5'-GCAAAGCACCAAGCCAAAC-3'	5'-GCATAGACACTTCCTTTGG-3'
PI-1	Solyc09g084470	5'-GAAACTCTCATGGCACGAA-3'	5'-CCTTCGCACATCAAGTTAGAG-3'
PI-2	Solyc03g020060	5'-CCTATTCAAGATGTCCCCGTTC-3'	5'-GGGCAATCCAGAAGATGG-3'

The genes are identified from the Sol Genomics Network (http://solgenomics.net/)

Supplemental Table 4. Plant phenotype of gene silenced plants

Genotype	Plant height (cm)	Leaf number per plant	Shoot fresh weight (g/plant)	Root fresh weight (g/plant)
pTRV	31.5	10.5	18.86	1.78
pTRV-*CCD7*	22.1	9.8	19.96	2.66
pTRV-*CCD8*	19.6	9.3	19.11	2.71
pTRV-*MAX1*	22.3	10.0	20.78	3.13

*Growth parameters were determined 48d after sowing. Data are the average of 6 plants.
