# Supplementary material for: Strigolactones positively regulate defense against root-knot nematodes in tomato
Source: J Exp Bot. 2018 Dec 21;70(4):1325–37. doi: 10.1093/jxb/ery439 (PMC6382333; doi:10.1093/jxb/ery439)
Supplement: Supplementary Figures S1-S8 [file ery439_suppl_supplementary_figures_s1-s8.pdf]

Figure S1

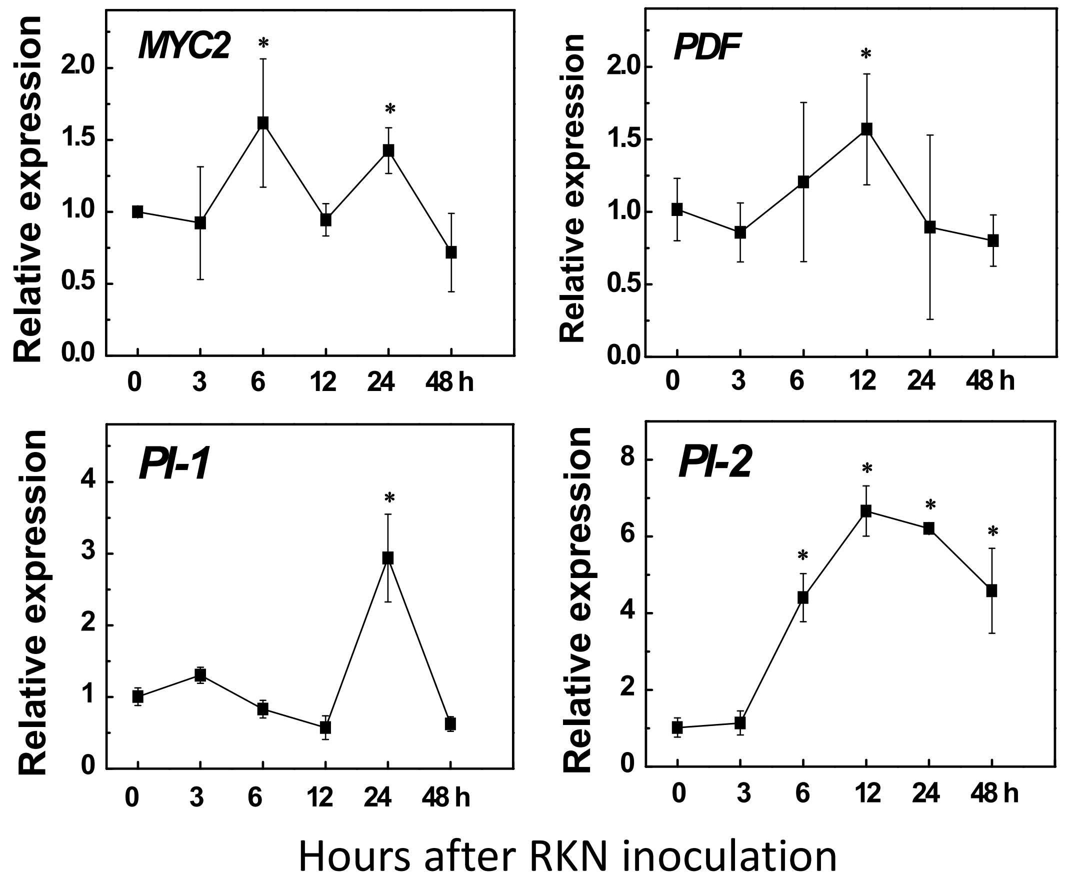

Figure S1. Time course of the transcript of defense related genes in roots of tomato after nematode infection. Data are the means of three biological replicates (±SD). The asterisks indicate significant difference as compared with values before RKN inoculation. Statistical comparisons were made using t-tests ($P < 0.05$).

Figure S2

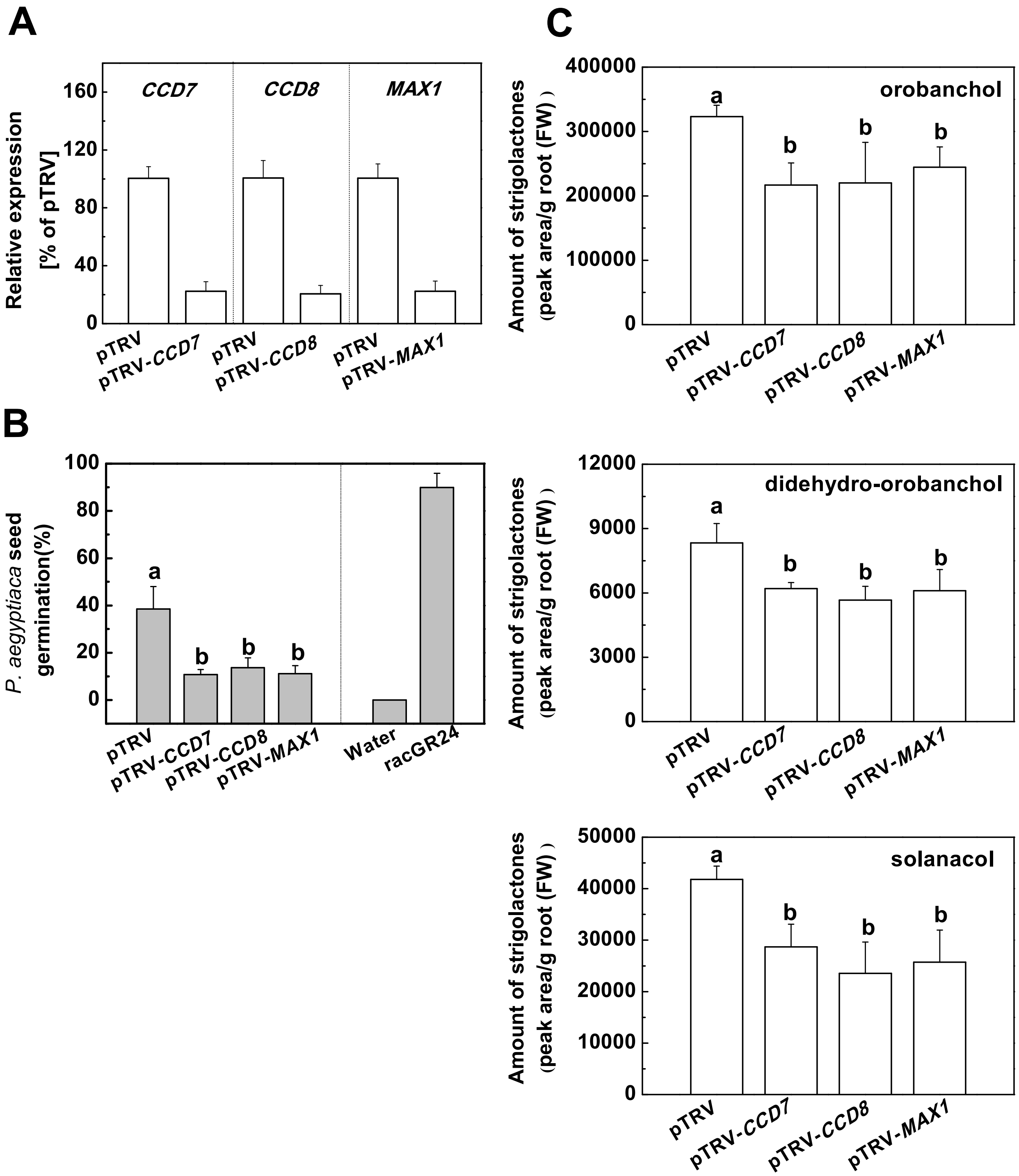

Figure S2. Silencing efficiency of SLs biosynthesis related genes in wild-type tomato roots. (A) Relative mRNA abundance of *CCD7*, *CCD8* and *MAX1* in VIGS plants**.** The levels were expressed as percentages of the mean levels in control pTRV plants which were defined as 100%. RNA was isolated from root samples collected 2 weeks after TRV infection, and transcript levels were determined using qRT-PCR. (B) *Phelipanche aegyptiaca* seed germination induced by the root extracts from *CCD7*-, *CCD8*- and *MAX1*-silenced plants compared to the empty vector pTRV plants. Sterilized distilled water as negative control while GR24 10^{-9} M solution as positive control**.** (C) Accumulation of SLs in the root extracts of VIGS plants. Data are the means of three replicates (±SD). Different letters above the bars indicate values that significantly differ (P<0.05).

Figure S3

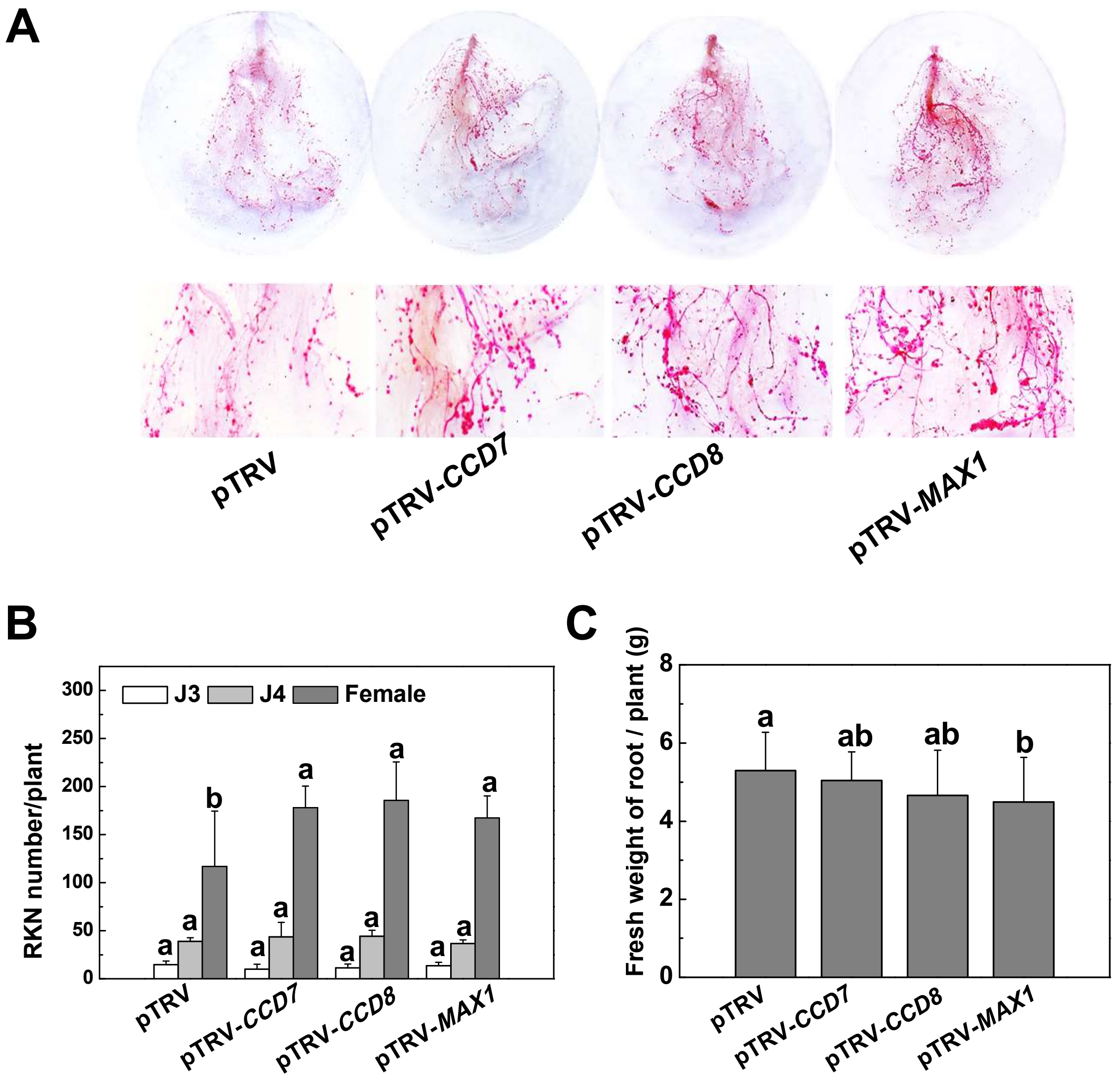

Figure S3. Influence of silencing of *CCD7*, *CCD8* and *MAX1* on the development of RKN and root weight of infected tomato plants. (A) Nematode galls observed after staining with acidified glycerin. (B) The number of RKN at J3, J4 and females in *CCD7*-, *CCD8*- and *MAX1*-silenced plants. Empty pTRV vectors served as controls. (C) Root weight in VIGS plants after RKN infection (n=18). Data were obtained from 20 plants 4 weeks after RKN infection (±SD). Different letters above the bars indicate values that significantly differ (P<0.05).

Figure S4

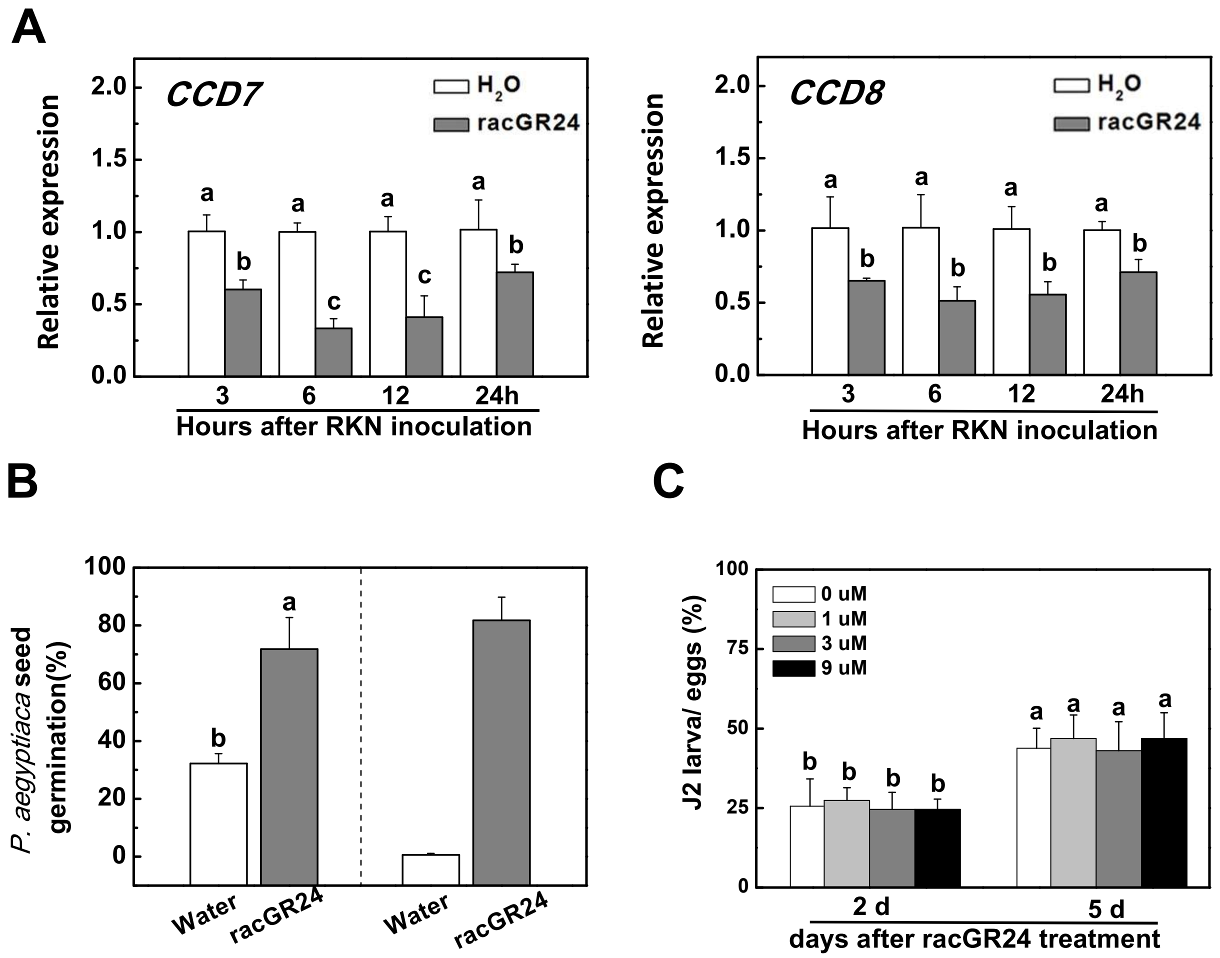

Figure S4. Effects of GR24 application on the expression of SLs biosynthesis genes and RKN growth. (A) Relative mRNA abundance of *CCD7* and *CCD8* in the roots of GR24-treated plants**.** RNA was isolated from wild type (Ailsa Craig) root samples collected 3h, 6h, 12h and 24 h after GR24 (3 μM) drenching. Distilled water containing equivalent concentration of solvent served as controls. Transcript levels were determined using qRT-PCR. (B) Seed germination of *Phelipanche aegyptiaca* as influenced by the root extracts from water or GR24-treated plants (left) or GR24 solution (right)**.** Root samples were collected 48 h after GR24 (3 μM) drenching. Sterilized distilled water as negative control while GR24 10^{-9} M solution as positive control (right). (C) Effect of different concentrations (1 μM, 3 μM, 9 μM) of GR24 solution on egg hatching of RKN (*Meloidogyne incognita*) after two and five days of incubation at 28°C. Data are the means of three replicates (±SD). Different letters above the bars indicate values that are significantly different (P<0.05).

Figure S5

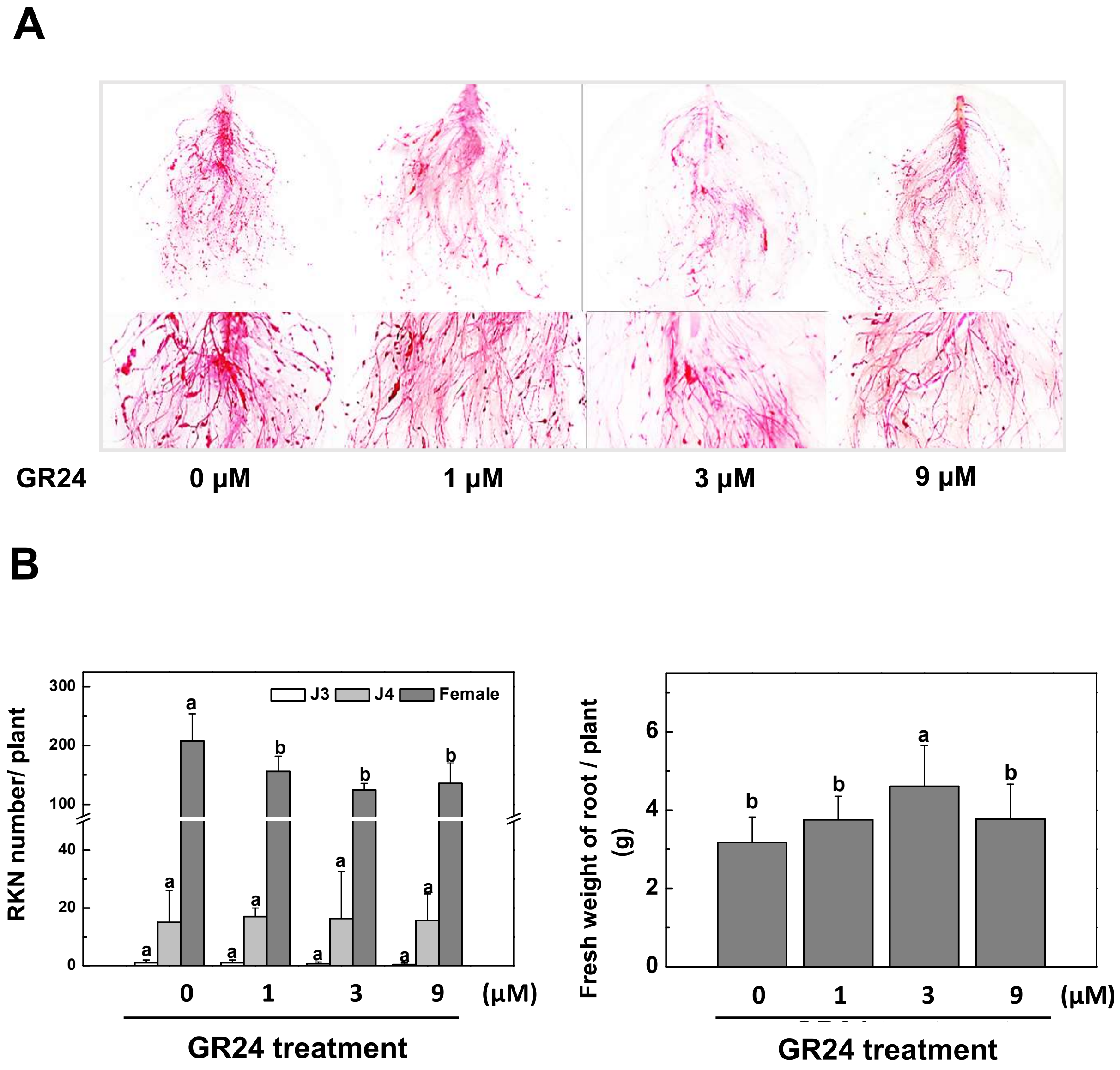

Figure S5. Influence of GR24 on the development of RKN and root weight in tomato plants. (A) Nematode galls observed after staining with acidified glycerin. (B) The number of RKN at J3, J4 and females in GR24-treated plants. (C) Root weight in plants after RKN infection. Distilled water containing equivalent concentration of solvent served as controls. Data were obtained from 18 plants 4 weeks after RKN infection (±SD). Different letters above the bars indicate values that significantly differ (P<0.05).

Figure S6

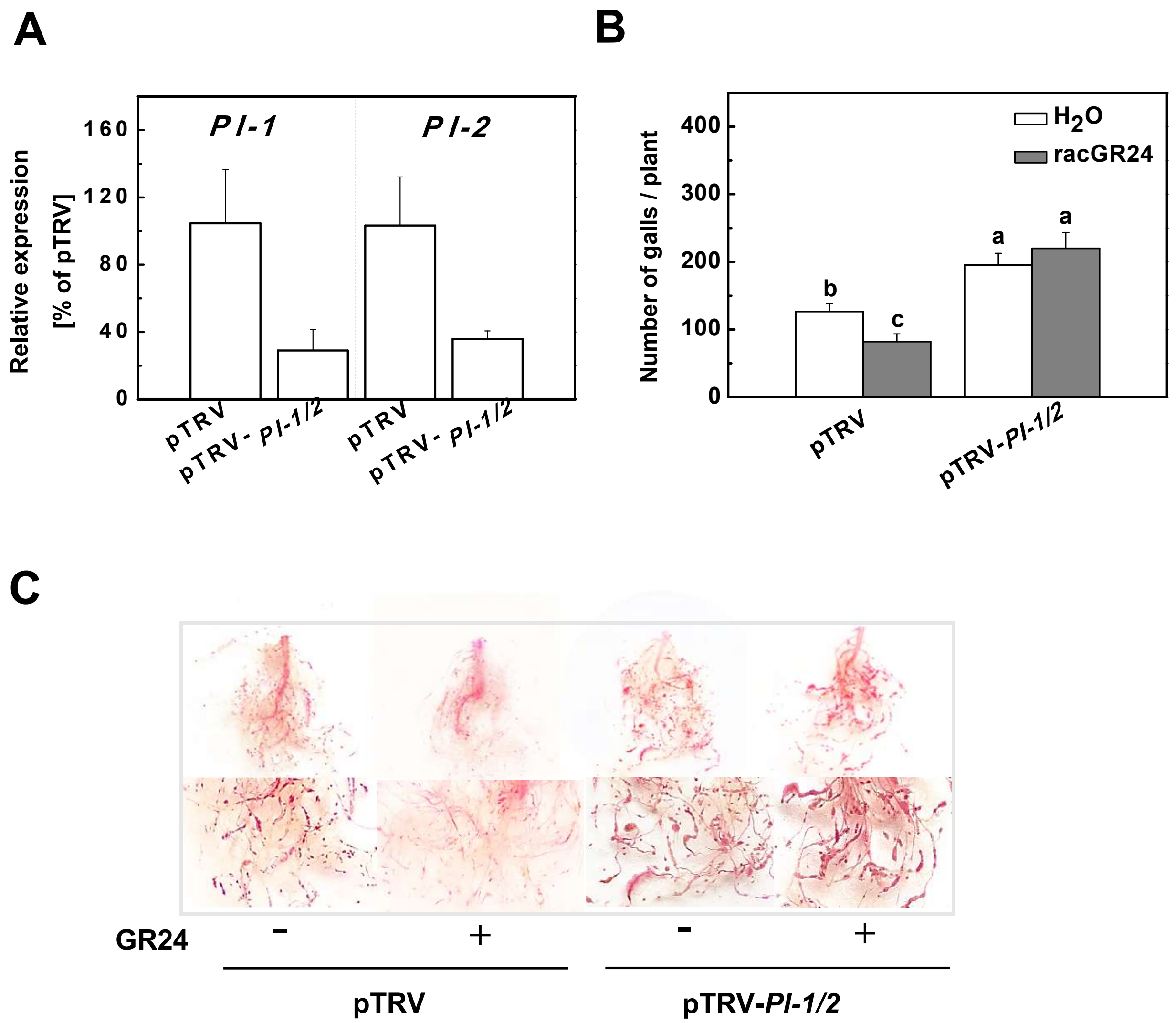

Figure S6. Effects of GR24 application on the RKN resistance of *PI-1/2* co-silenced plants. (A) Relative mRNA abundance of *PI-1* and *PI-2* in VIGS plants**.** The levels were expressed as percentages of the mean levels in control pTRV plants which were defined as 100%. RNA was isolated from root samples collected 2 weeks after TRV infection, transcript levels were determined using qRT-PCR. (B) The number of RKN induced root galls per plant in *PI-1/2* co-silenced plants with or without GR24 application. Empty vector pTRV is negative control. GR24 at 3 μM was applied 24 hours before RKN infection, and distilled water containing equivalent concentration of solvent served as controls. (C) Nematode galls observed after staining with acidified glycerin. Galls number was calculated 4 weeks after RKN infection. Thirty plants per treatment were used in each experiment. The RKN experiment was repeated three times, and similar results were obtained each time. Data from one representative experiment are presented. Data are the means of three replicates (±SD). Different letters above the bars indicate values that are significantly different (P<0.05).

Figure S7

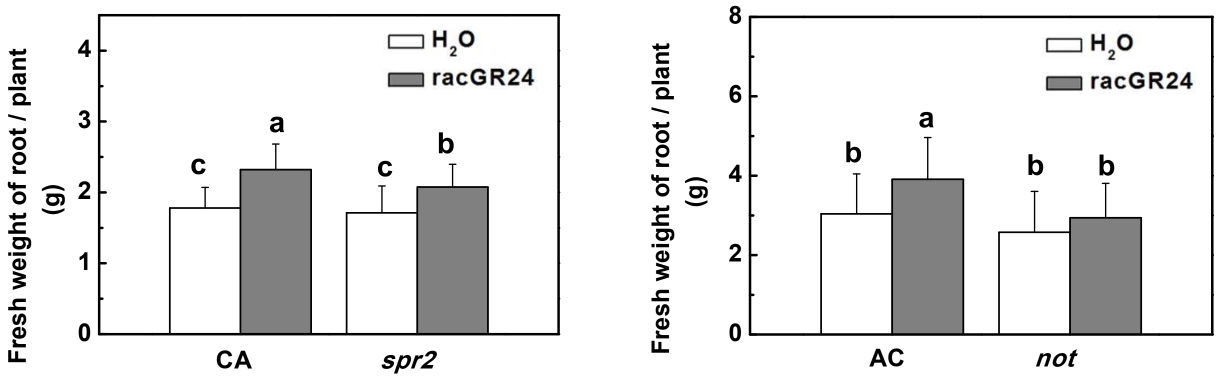

Figure S7. **Root weight in WT, *spr2* and *not* plants after RKN infection.** GR24 at 3 μM was applied 24 hours before RKN infection. Distilled water containing equivalent concentration of solvent served as controls. Data were obtained from 18 plants 4 weeks after RKN infection (±SD). Different letters above the bars indicate values that significantly differ (P<0.05).

Figure S8

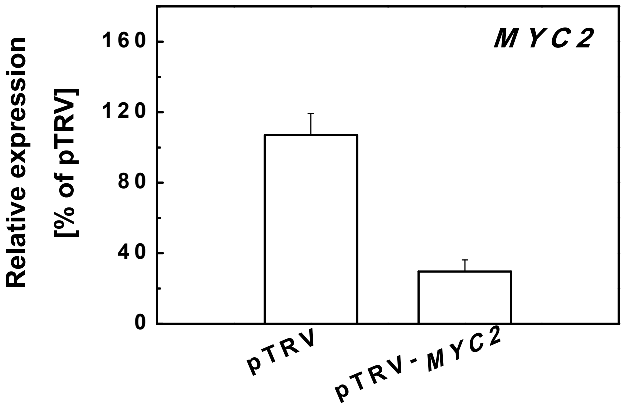

Figure S8. Silencing efficiency of *MYC2* in wild-type tomato roots. The levels were expressed as percentages of the mean levels in control pTRV plants which were defined as 100%. RNA was isolated from root samples collected 2 weeks after TRV infection, transcript levels were determined using qRT-PCR. Data are the means of three replicates (±SD).
